# Empowering Underrepresented Minority Students: a STEM-Focused Research Internship Program Bridging the Path to Graduate and Professional Success

**DOI:** 10.1007/s10755-025-09797-x

**Published:** 2025-04-12

**Authors:** Ihsan Elkhider, Nishika T. Edwards, Richard L. Goodwin, Renee J. Chosed, Larry Lenard Lowe, Steffani Driggins, Randall H. Harris, Kimberly Shorter, Zhi Gao, Stephen Ojo, Olukayode Karunwi, Nnenna Igwe, Thomas I. Nathaniel

**Affiliations:** 1 Biomedical Sciences, University of South Carolina School of Medicine Greenville, Greenville, SC 29605 USA; 2 Biomedical Engineering, University of South Carolina, Columbia, SC USA; 3 Biology, Benedict College, Columbia, SC USA; 4 Biology, Claflin University, Orangeburg, SC USA; 5 Biology, University of South Carolina Upstate, Spartanburg, SC 29303 USA; 6 Bioengineering, Clemson University, Clemson, SC USA; 7 College of Engineering, Anderson University, Anderson, SC 29621 USA

**Keywords:** Internship, Research, Underrepresented Minorities (URMs), Mentors

## Abstract

The goal of this Research Education Program (REP) is to increase diversity in science, technology, engineering, math, and medicine (STEMM) fields. However, few programs include research internship opportunities and summer education programs for student participants. In our REP we integrated a research internship program into the summer curriculum and provided stipends aimed to alleviate the financial stress for underrepresented minority (URM) students, allowing them to focus on research, academics, and professional development. More than 90% of research mentors were satisfied with interns’ ability in various aspects of STEM research, including integrating research with academic work, developing research skills, engaging in research, formulating scientific hypotheses, implementing constructive feedback, and their readiness to become independent researchers. Over 80% of student interns reported satisfaction with the positive impact of the internship program on their professional development in scientific research, oral presentation, and written communication skills. This improvement was attributed to experiences such as preparing posters and manuscripts, collaborating in teams, working independently, and engaging in overall program activities. There was a significant positive association (r(9) = .60, *P* < 0.05) between student satisfaction and research mentors' evaluation of their satisfaction with students' performance during the research internship program.

## Introduction

The underrepresentation of minorities (URMs) in science, technology, engineering, math, and medicine (STEMM) fields is an important issue. Existing studies (Bhatti et al., 2021) show that Black and Hispanic professionals are underrepresented in STEM jobs in the overall U.S. workforce. This disparity also impacts the quality and the degree of research in the STEMM fields (Clayborne et al., 2021). URM students face many challenges in pursuing STEMM education including stereotype threat, impostor phenomenon, and lack of social connectedness that disproportionately affect URM students in majority-dominated fields (Markle et al., 2022). These challenges and other personal circumstances can discourage many students from applying to graduate programs in STEMM.

The STEMM job market is projected to grow by 8.8% between 2017 and 2029 (Palid et al., 2023), a rate much faster than the 3.7% projected growth for all other occupations (Palid et al., 2023). This growth represents a significant opportunity, but without addressing the challenges faced by URM students, the racial disparity gaps within STEMM will likely persist. Efforts to increase diversity in STEMM must address these challenges directly. Action items could include providing more supportive academic resources, implementing structured mentoring networks (Montgomery, 2017), and creating an inclusive environment that counters microaggressions and stereotype threats.

The National Institutes of Health (NIH) is taking several important steps to address the racial disparity gaps within STEMM through different initiatives including the NIH R25 Research Education Program (REP). This program not only supports URMs in completing their bachelor’s degree but also supports their transition into the STEMM pipeline. The NIH REPs primarily target URM undergraduate and graduate students interested in graduate school and STEMM research careers. The primary goal is to bolster the preparedness of URM scholars for research-intensive education, thereby maximizing their chances of success and persistence in STEMM careers. The initiative aims to address the lack of diversity at the faculty and senior scientist level, which is a significant step towards creating a more inclusive and diverse STEMM field.

URMs in STEM research internship programs have mainly focused on African Americans (Blacks), Hispanics/Latinos, American Indians/Alaska Natives, Native Hawaiians, and Pacific Islanders (Moreira et al., 2019). This is because these groups are less represented in STEM fields compared to their proportion in the general population. The reason is often attributed to barriers to accessing high-quality research internship opportunities. Implementing research internship activities throughout the academic year provides both academic enrichments along with peer and/or faculty support and is therefore part of the STEM opportunity structure. Such an opportunity has been reported to be crucial for URMs as they provide students with the chance to build their network, find and connect with mentors, gain research experience, and receive financial support (Hinton et al., 2020; Shuler et al., 2021). Many studies have shown that involving URM students in research internships may allow students to feel like they are making meaningful contributions to the field, learning the norms of science, and decreasing feelings of marginalization by integrating the students into the academic and STEM community (Hurtado et al., 2009; Marriott et al., 2021). However, few REP programs integrate research internships into their training programs (Dockry et al., 2022; Michel et al., 2021). We address this challenge by incorporating research internship activities into our REP program. The research internship activities are implemented throughout the academic year in addition to the summer research program of our NIH R25 program.

In general, undergraduate research opportunities represent a mechanism for recruiting and retaining URMs with the potential for careers in different STEM fields (Beasley et al., 2024; Bruthers et al., 2021; Castro, 2012; Schmidt et al., 2020). At the National level, the National Institutes of Health’s Maximizing Access to Research Careers (MARC) (Hall, 2017) and the National Science Foundation’s Louis Stokes Alliances for Minority Participation (LSAMP) (Gates et al., 2022) promote undergraduate research programs, that combine mentoring, providing financial assistance, to participate in conferences, and research experience for URMs. In addition, the Behavioral Research Advancements in Neuroscience (BRAIN), an undergraduate research program that utilized collaborative learning models that provides a larger collaborative laboratory environment for URMs with several instructors to provide research skills and increase participation in research programs (Frantz et al., 2017). Although undergraduate research internship opportunities for URMs vary in quality and type, they are generally marked by engagement in research practices, collaboration and teamwork, iterative refinement, mastery of research techniques, reflection on issues and work, communication of results, and structured mentorship to improve research skills for URMs (Council, 2011). In general, undergraduate research internship opportunities help improve crucial skills, including experimental design, data management, communication, and networking among well-represented and URM groups (Bruthers et al., 2021). The skills gained in undergraduate research internship experiences further persist into graduate school (Gilmore et al., 2015). URMs are particularly impacted by gaining a scientific identity, dependent on solid mentor relationships, and customizing program goals to enhance their research skills. Therefore, a REP that successfully promotes inclusion in the sciences must not only focus on skill building but must also consider the unique “experiences and perceptions” of URM individuals and research mentors who participate in the research internship programs. The impact of such programs on URMs and their perceptions of research is indeed a valuable area of study. The unique experiences of URMs can provide insights to further refine and improve these programs and make STEM fields more accessible, and inclusive, and promote scientific identity.

### Theoretical Framework

Our theoretical framework is that a successful scientific graduate preparation REP program must not only develop research skills but must also enrich the scholar’s “science identity,” and that this includes knowledge of the STEMM discipline, ability to perform relevant scientific research, as well as a sense of belonging and recognition within a scientific network (Carlone & Johnson, 2007). Our theoretical framework emphasizes that a successful graduate preparation Research Experience Program (REP) goes beyond just equipping scholars with research skills. It also nurtures their "science identity”. Our view is that science identity is how individuals perceive themselves in relation to science. This includes their sense of belonging, competence, and recognition within scientific disciplines. This identity plays an important role in influential a person's engagement, persistence, and success in science-related fields. This identity is shaped by recognition, mentorship, belonging, and representation**.** When individuals see themselves reflected in STEM fields, they are more likely to persist and thrive. We believe that fostering a strong science identity can lead to greater persistence in STEM careers, particularly for women and marginalized groups. Our mentorship program plays a crucial role in fostering a strong science identity**,** especially for students or early-career individuals who might not yet see themselves as "science people. Our approach positions our REP programs not only as skill-building platforms but also as transformative spaces that prepare scholars to thrive as confident, innovative contributors in STEMM fields.

This is because URMs will experience the collaborative and empowering culture of science, exhibit strong science identities and high self-efficacy, through their ability to conduct rigorous scientific research, satisfaction with their research experience during the research internship, and present their work at national conferences to confer a sense of belonging and recognition within the scientific network. Therefore, our REP includes a mentored research internship experience but also directly provides opportunities to hone scientific skills and gain experiences outside the laboratory that are necessary components of success in scientific careers. These experiences occur within the context of a cohort of scholars who are intentionally provided with opportunities to build community both within the cohort and within the broader research environment at our institution, local, and national conferences, and institutions participating in the program. Through this program, scholars gained increased self-efficacy in a spectrum of competencies, thereby fostering greater science identity and a heightened inclination to pursue and persevere in scientific research careers.

### Research Questions

Similar to the overarching goal for all NIH-funded REPs, our objective was to establish a program aimed at increasing the number of URM scholars who matriculate in competitive STEMM graduate programs and to prepare these scholars for subsequent success within those programs thereafter. We postulated that a research internship that provides URMs with financial support provides one-on-one training in various laboratory techniques, develops skills in critical thinking, problem-solving, and data analysis, and allows them to present their work at research conferences will provide skills and readiness to become independent in conducting research. In addition, it will provide a less arduous process of developing a science identity especially when they are provided with the opportunity to present their research findings at national conferences and receive validation and recognition from scientists at the national level. Moreover, research mentors can significantly impact a URM’s developing identity by acting as crucial role models and guides, influencing their research approach, providing constructive feedback, and positively promoting their career trajectory, essentially helping them define their "research identity" through mentorship and guidance, resulting in positive perception and satisfaction of the research mentees. Therefore, URMs could see themselves as scientists and belonging to science. This sense of belonging, positive perception and satisfaction are critical in maintaining motivation through interpersonal challenges on top of demanding science education. Therefore, the following research questions guided our study:

- Research question 1: Are URMs and their research mentors satisfied with how the internship program improved URMs professional development in scientific research?
- *Hypothesis*; More URMs and research mentors will be satisfied with how the internship program improved URMs professional development in scientific research, oral presentation, and written communication skills through preparing their posters and manuscripts.
- Research question 2: Is there a significant correlation between student satisfaction and research mentors' evaluation of their satisfaction with students' activities during the research internship program?
- *Hypothesis*: There is a statistically significant correlation between student satisfaction and research mentors' evaluation of their satisfaction with students' activities during the research internship program.

### Rationale for this Study

Several efforts have specifically attempted to provide students with the tools needed to acquire both academic and practical knowledge leading to scientific research careers through Fall and Spring research internship research opportunities, while further providing URMs and their research mentors with financial assistance to support the research internship activities. Common assumptions regarding racial and ethnic inequality in research focus on a perceived lack of satisfaction in URMs resulting in low motivation and lack of preparation for a research career. This depends on whether URMs are satisfied with how the research internship or summer research program improves their professional development in scientific research, or whether the research mentors are satisfied with URMs performance during the research internship program.

Providing URM undergraduate students with research opportunities significantly increases their likelihood of staying in science majors, considering graduate school, and ultimately pursuing careers in science (Sellami et al., 2021). In this study, we provided research internship experience within REP and created a research environment grounded in more collaborative teamwork to facilitate a culture of science in such a manner that comprehensively supports URMs. The research internships are closely aligned with students' goals, particularly those aspiring to pursue graduate programs in STEMM fields. The approach provides a key strategy to motivate URM to find the tools they need, acquire knowledge about the scientific method, as well as enhance students’ interest in becoming scientists and we examined URM student experiences in these programs.

### Importance of Implementing a Research Internship Program within a REP Program

Initially, the program focused on building a strong support network and familiarizing scholars with the environment of summer research and academic enrichment activities. Additionally, it provided professional development training in essential skills and guided scholars through the graduate program application and admissions process. We expanded the program to provide a full-time, year-round research internship aimed at providing comprehensive scientific training and developing skills crucial for success in science, which are not typically taught in the research laboratory. For example, preparing a poster, scientific writing, and presenting oral and posters at conferences. As a result, we developed a research internship program that integrates laboratory training, support in a multitude of relevant professional conference presentations and proficiencies, and deliberate community-building among scholars with a scientifically trained program. We provided a summer research opportunity and extended the research opportunity throughout the academic year in the third year of implementing the program with a financial incentive for participating in the internship program. While it could be challenging to combine academic work with research commitment during the academic year, exposure to authentic research experiences for URMs during this time has been demonstrated to enhance persistence (Rodenbusch et al., 2016). Therefore, we investigate whether an intervention of this type would be perceived positively by both the interns and research mentors. In this study, we present findings and outcomes of students' and research mentors' perceptions of the research internship program during the implementation of our NIH R25 program. Our findings will reveal the importance of implementing a research internship program within a REP program. In addition, it will highlight how a research internship that provides professional development in scientific research, oral presentation, and written communication skills through preparing posters and manuscripts promotes URMs experience and culture of science, career goals as a result of conducting scientific research, and promoting scientific identity.

## Methods

### The Study Population

A total of 17 URM students participated in the summer REP program: African American (50%), Asian (15%), Caucasian (20%), Hispanic (5%), and Mixed Race (10%). One of the mixed-raced students identifies as both African American and Hispanic. Others identify as both African American and Caucasian. Of the student cohort, 75% are from a socioeconomically disadvantaged community which includes lower education rates, lower income, homelessness, and foster care at some point. 80% of the students identified as female, while 20% of students identified as male. Participants included students majoring in the following disciplines: Biology, Biochemistry, Bioengineering, Public Health, Exercise Science, and Kinesiology. Table 1 summarizes the demographics of the participants including institutions of participants (HBSCU or majority) and based on Carnegie classification.

**Table 1:** presents a summary of participants in the program including sex, race/ethnicity, undergraduate institutions, and admissions to graduate programs

Scholar demographics	*n* (total = 47)	Percent
Female	8	32
Male	17	68
Black/African American	16	64
Hispanic/Latino(a)	1	5
White/Caucasian	3	15
Asian	3	15
Mixed Race	2	10
Participants that attended primarily undergraduate institutions		
Participants that attended minority institution (HBSC)	9	36
Participants that attended majority institution	16	64
Carnegie classification		
Baccalaureate colleges: diverse fields	4	16
Master’s colleges and universities (larger program)	10	40
Baccalaureate colleges: arts and sciences	4	16
Research universities (very high research activity)	3	6
Research universities (high research activity)	4	9
Master’s colleges and universities (medium program)	11	45
Admission to graduate programs during REP		
Not successful	27	57
Successful	20	43

### Description of the Summer Research Education Program

The goal of the Summer REP program is to provide professional development activities and expose URMs to scientific, biomedical, behavioral, and public health academic training and research that will aid in preparing them for the next phase of their educational and professional careers. The program provides educational activities that encourage URMs in STEM programs to pursue further academic studies or careers in scientific research. The research activity component of the program focuses on improving URMs' research skills, and readiness for career advancement in STEM programs. Participants were recruited from South Carolina colleges and universities specializing in STEM fields through social media sites, flyers, brochures, virtual meetings with probable research mentors, word-of-mouth, and targeted recruitment from research mentors at collaborating colleges and universities with a 100% response rate. Successful admission and acceptance into the REP program required the following components: 1). A student having a URM status, enrollment as a sophomore, junior, or senior at a college or university in South Carolina, a GPA of 3.0 or higher, and holding a U.S. citizenship or valid Green card. 2) Completion of an NIH R25 summer application that included the application, college transcript, personal statement, and two letters of recommendation 3). Successful completion of an interview with faculty and Program Coordinator 4). Receipt of signed REP acceptance letter. Following acceptance, each student was matched with a research mentor who was closely aligned to their major or area of research interest as well as their location within the state.

Recruitment in the first year of the program in 2020 was slow as we were only able to recruit 7 students to participate in the program at the University of South Carolina since most participants did not want to travel for on-campus implementation of the program. To address these problems in the second year of the program, we created partnerships with non-historically and historically black colleges and universities (HBCUs) in South Carolina, Anderson University, Benedict College, Claflin University, Clemson University, University of South Carolina Upstate, and North Greenville University. This allowed us to recruit more students (20 students) and more research mentors to participate in the program in the partnership institutions. In the third year, we recruited 30 students who participated in the program and then implemented the research internship program for this cohort of students. Table 1 presents a summary of participants in the program including sex, race/ethnicity, undergraduate institutions, and admissions to graduate programs.

**Fig. 1:**
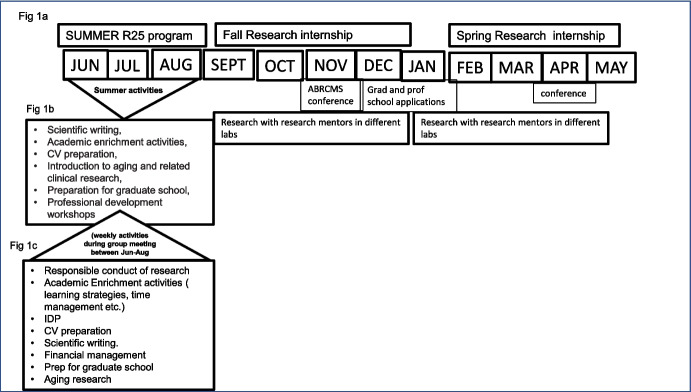
Description of the implementation of the NIH R25 REP program

The implementation of the NIH R25 REP program is presented in Fig. 1. The first iteration of the REP program starts each year in early June and ends in late July. Each day's activity is divided into morning and afternoon sessions. Morning sessions included different components: scientific writing, academic enrichment activities, CV preparation, introduction to aging and related clinical research, preparation for graduate school, and professional development sessions in financial literacy and emotional management (see Fig. 1b). Morning sessions end at 11:00 am Monday to Friday, while students dedicate afternoons from 1:00 pm to 4:00 pm for their research work in their various labs.

**Fig. 2:**
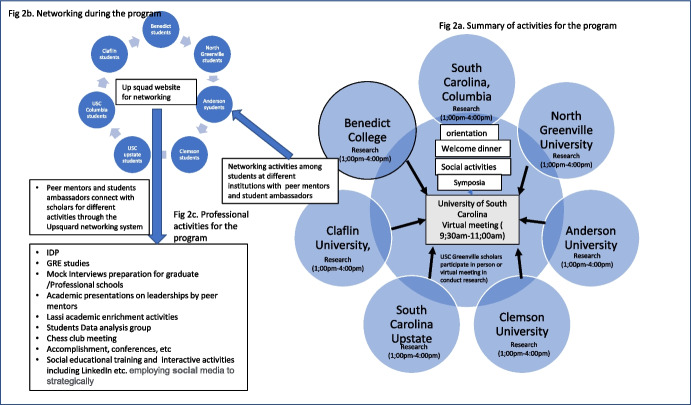
Summary of the activities for the program

Apart from the morning sessions that were implemented virtually and research activities that were implemented in-person at different institutions, scholars also had the opportunity to visit the University of South Carolina School of Medicine Greenville during the orientation session, welcome dinner, and at least 2–3 other times for social activities and during the end of summer symposia. A major goal of the REP was to build community among the cohort and between the scholars and mentors at different institutions. Therefore, an active online App was utilized (Upsquad) within and between the partnering schools for social and professional networking (Fig. 2B). This App facilitated the peer mentoring and ambassadors’ social interactions with scholars during the program in addition to the in-person meetings during their campus visits. This App allowed peer mentors, ambassadors, and faculty mentors to socially interact with scholars. Professional interactions among these groups also occurred through educational activities (Fig. 2C) including, IDP, mock interviews for graduate or professional schools, expert panel sessions, etc.

### Description of the Research Internship Program

The program concluded with an annual symposium where the students delivered oral and poster presentations. After the summer program, students continued to receive mentorship through monthly meetings and attendance at national conferences. In addition, the REP provided continued support for URMs beyond the duration of the summer NIH R25 program. This includes practical research internship experience, networking opportunities, and potential authorship on publications. For the research internship program, each research mentor provided personalized one-on-one mentoring to help interns navigate the research process and develop professionally. Each research mentor met with each scholar and assessed the student's ability to combine the research internship with their academic work. This was a major factor in selecting scholars to participate in the internship, and more than 90% of students who participated in the summer program continued with the fall research internship program. The internships started in September and ended in December, while the spring internships started in January and ended in April. The ability to continue with the spring internship was based on progress which was evaluated based on the submission of a poster and, or a conference abstract. In addition, each research mentor was provided with a questionnaire to evaluate each scholar. The positive evaluation of the scholar and submission of a poster or conference abstract was a major criterion considered for the renewal of the internship for the spring internship for each scholar. All students who participated in the fall internship qualified and participated in the spring internship research program. Students were paid to work for between 15–20 h per week during the research internship program. Interns worked in a research group directed by their research mentors. Interns were offered research opportunities in the biomedical, behavioral, and data sciences with opportunities to explore basic, translational, and clinical research in aging. Responsibilities of each research intern included participation in different aspects of the research process, including literature reviews, data collection and analysis, experimental design, programming, writing abstracts or manuscripts, and presenting findings at meetings or conferences.

#### Evaluation

The systematic collection, review, and evaluation of assessment data was focused on different levels utilizing Kirkpatrick’s four-level evaluation model (Rouse, 2011). In this model, level 1 focuses on learner satisfaction or reaction to the program (i.e. student’s perception of the quality of their learning), while level 2 evaluates changes in learner behavior in the context for which they are being trained (application of the learned knowledge). Level 3 focuses on the program’s results in its larger context (overall success of the program). In this study, we evaluated our data based on Kirkpatrick Model's first two levels, focusing on participants' reactions to the training (Level 1) and the actual learning outcomes achieved (Level 2).

The first phase of the evaluation using Level 1 Kirkpatrick model focused on students' perceptions of the internship program. Students completed a confidential seven-item questionnaire. They rated their level of satisfaction with how the internship program improved their professional development in scientific research, improved their oral presentation skills, their level of satisfaction of how the internship program improved their written communication skills (i.e. posters, manuscripts), and how the internship program improved their ability to work in a team. We also evaluated their level of satisfaction with how the internship program improved their ability to work independently, their satisfaction with the internship program's overall activities, and how the internship program helped them to participate in local, regional, and national conferences. Responses were rated using a five-part Likert scale: very dissatisfied (1) to very satisfied (5).

The second phase (Level 2) assessed the extent to which participants have acquired new knowledge, skills, or attitudes as a result of the program. The research mentors completed a confidential nine-item assessment. The first section of the instrument required the mentors to complete nine questions to assess each intern’s performance during the research internship program and determine their eligibility for renewal to participate in the spring program. The focus was on the research performance of each research intern whether working independently or as part of a research team. This included assessing their ability to balance academic work and research responsibilities, for example, managing time efficiently and seamlessly integrating research with academic work during the internship. Additionally, the evaluation aimed to gauge the improvement of mentees' research skills over the course of the internship program. Other parameters evaluated included intern’s engagement and productivity throughout the research internship program. This encompassed various aspects such as their acquisition of new scientific skills, proficiency in experimental design, as well as data collection and analysis. Additionally, we assessed the intern’s ability to use constructive feedback in making positive changes related to research such as experimental designs, etc. Furthermore, we evaluated their readiness to autonomously conduct research, exemplified by their capability to identify research questions and devise experiments with minimal supervision during the internship. A five-part Likert scale was used to rank the items from very dissatisfied (1) to very satisfied (5).

### Data Analysis

Statistical Package for Social Sciences v. 26.0 for Windows (SPSS, Chicago, IL) was used for statistical analysis. Evaluation of the participants was done using descriptive statistics collected from the scaled responses. Responses were distributed using percentage calculations for the satisfaction of interns by their respective mentors on their ability to combine research with academic work, improvement of interns' research skills and ability of the intern to learn new scientific skills, design of experiments, data collection, and analysis. We also analyzed data on the satisfaction of the research mentors on each intern’s productivity in terms of the ability to conceptualize and formulate scientific hypotheses and test them during data collection, use constructive feedback in making positive changes related to research including experimental designs etc. Lastly, we assessed the interns' readiness to autonomously conduct research. Students' perceptions of the internship program were analyzed using percentage calculations for the level of satisfaction of how the internship program improved their professional development in scientific research, oral presentation skills, written communication skills, working in a team, working independently, overall activities, and helping in participation in local, regional, and national conferences. We determined the correlation between research mentors' satisfaction with the productivity of each intern and the interns' satisfaction with their productivity. The productivity variables were extracted from (productivity in research performance, research skills, engagement with the team communications or presentation skills, etc.) as assessed by research mentors and students' satisfaction with their productivity during the internship. This includes productivity in research skills or performance, presentation skills, improved written communication skills, ability to work in a team, and independently. This allowed us to describe the relationship between URM satisfaction and mentors satisfaction with their productivity during the internship program.

## Results

Table 2 presents results from research mentors (*n* = 14 for Fall and Spring) following the evaluation of interns during their participation in the program. 79% of research mentors were very satisfied, while 21% were satisfied with the performance of their interns by working independently or with their team in the laboratory during the internship. 71% were very satisfied while 29% were satisfied with their interns' ability to combine research with academic work during the internship, 57% were very satisfied and 43% were satisfied with the improvement of their mentee’s research skills during the internship program. 50% were very satisfied and satisfied with their interns' engagement in research during the research internship program, while 93% were very satisfied, and 7% were satisfied with the ability of their interns to conceptualize and formulate scientific hypotheses and test them during data collection. Interns' ability to use constructive feedback in making positive changes related to research including experimental designs recorded 79% of very satisfied and 21% satisfied, while interns' readiness to become independent in conducting research received 64% of very satisfied and 43% satisfied of research mentors satisfaction.

**Table 2:** Evaluation of the participants by mentors at the end of the first phase of the program 2

		Fall 2023					Spring 2024				
		(1) V. Diss	(2) Diss	(3) Neu	(4) Sat	(5) VSat	(1) V. Diss	(2) Diss	(3) Neu	(4) Sat	(5) VSat
Q1	Rate the research performance of your mentee either by working independently or with your team in the lab during the internship	0%	0%	0%	21%	79%	0%	0%	0%	0%	100%
Q2	Rate the mentee’s ability to balance between academic work and research, for example, managing time efficiently and being able to combine research with academic work during the internship	0%	0%	0%	29%	71%	0%	0%	0%	18%	82%
Q3	Rate the improvement of your mentee’s research skills during the internship program	0%	0%	0%	43%	57%	0%	0%	0%	45%	55%
Q4	Rate your mentee’s engagement in research and collaboration during the research internship program	0%	0%	0%	50%	50%	0%	0%	0%	36%	64%
Q5	Rate your mentee’s engagement and productivity during the research internship program, for example, learning new scientific skills, design of experiment, data collection and analysis	0%	0%	0%	7%	93%	0%	0%	0%	30%	70%
Q6	Rate your mentee’s ability to use constructive feedbacks in making positive changes related to research including experimental designs etc	0%	0%	0%	21%	79%	0%	0%	0%	9%	91%
Q7	Rate your mentee’s readiness to becoming independent in conducting research, for example, was able to identify research questions and design experiments with little supervision during the internship	0%	0%	0%	43%	64%	0%	0%	0%	35%	65%

Very dissatisfy (V.Diss), Neutral (Neu), Satisfy (Sat), Very satisfy (Vsat)

**Fig. 3:**
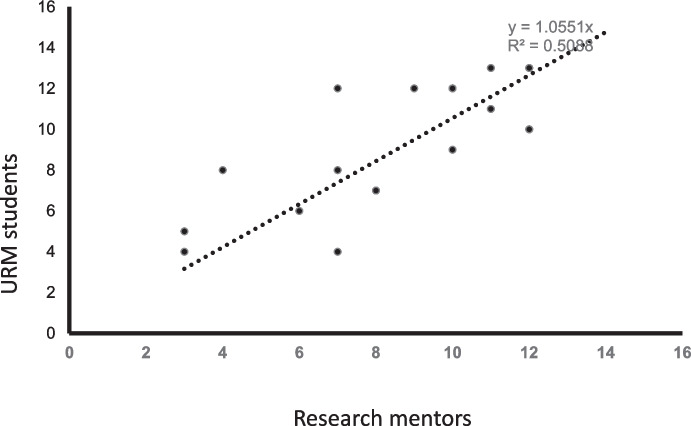
Association between student satisfaction and research mentors' evaluation of their satisfaction with students' activities during the research internship program

Table 3 presents the distribution of responses (*n* = 18 for Fall and Spring) from the students. 72% of interns were very satisfied, and 28% were satisfied with how the internship program improved their professional development in scientific research. 50% were very satisfied with how the internship program improved their oral presentation skills, while 44.44% were satisfied, 0.62% were neutral. 66.67% were very satisfied, while 22.22% were satisfied and 11.11% were neutral about how the internship program improved their written communication skills through the preparation of their posters and manuscripts. 61.11% believed and were very satisfied, while 22.22% were satisfied and 16.67% were neutral about how the internship program improved their ability to work in a team. 83.33% were very satisfied, while 16.67% were satisfied with how the internship program improved their ability to work independently. 66.67% were very satisfied, while 33.33% were satisfied with how the internship program's overall activities. 61% were very satisfied, 28% were satisfied and 11% were neutral on whether the internship program helped them to participate in local, regional, and national conferences. Results of the Pearson correlation indicated that there was a significant positive association (r(9) = 0.60, *p* < 0.05012) between student satisfaction and research mentors' evaluation of their satisfaction with students' activities during the research internship program(Fig. 3).

**Table 3:** Participants evaluation of the program fall and spring

		Fall 2023					Spring 2024				
		(1) VDiss	(2) Diss	(3) Neu	(4) Sat	(5) VSat	(1) V. Diss	(2) Diss	(3) Neu	(4) Sat	(5) VSat
Q1	Rate your level of satisfaction of how the internship program improved your professional development in scientific research	0%	0%	0%	28%	72%	0%	0%	0%	28%	72%
Q2	Rate your level of satisfaction of how the internship program improved your oral scientific presentation skills	0%	0%	0.62%	44%	50%	0%	0%	0%	42%	58%
Q3	Rate your level of satisfaction of how the internship program improved your written communication skills (i.e. Poster/manuscript)	0%	0%	11%	22%	67%	0%	0%	0%	42%	58%
Q4	Rate your level of satisfaction of how the internship program improved your ability to work in a team	0%	0%	17%	22%	61%	0%	0%	0%	40%	60%
Q5	Rate your level of satisfaction of how the internship program improved your ability to work independently	0%	0%	0%	17%	83%	0%	0%	0%	42%	58%
Q6	Rate your level of satisfaction of how the internship program's overall activities	0%	0%	0%	33%	67%	0%	0%	0%	30%	70%
Q7	Rate your level of satisfaction of how the internship program helped you to participate in local, regional, and national conferences	0%	0%	11%	28%	61%	0%	0%	0%	43%	57%

Very dissatisfy (V.Diss), Neutral (Neu), Satisfy (Sat), Very satisfy (Vsat)

## Discussion

Among the different activities within the URM students STEMM educational training programs, one of the most widely reported activities in the education training program literature is undergraduate research (Eagan et al., 2013; Edwards et al., 2024; Estrada et al., 2016). A major component of any URM education research program is the integration of URM students into the culture of a research career (Estrada et al., 2016, 2018; Jehangir et al., 2022). The goal is to promote URM students’ confidence and skills to improve their capability to apply their understanding of science in an experiential setting (Ballen et al., 2017; Michel et al., 2021). Particularly, it increases the probability of completing their degree, and graduation with a strong GPA (Sellami et al., 2021), and ambitions to pursue a STEM career or graduate degree (Eagan et al., 2013).

We observed that a significant number of research mentors were very satisfied with the research performance of their URM student interns. In evaluating the research performance of their interns, mentors considered the posters, or abstracts submitted to local or national conferences as the major outcome. All the participants in the summer REP program continued with their research in the fall internship, and all participants submitted at least one abstract for local or national conferences. This is also reflected in the high rate of satisfaction in the engagement and productivity of their interns during the research internship program. For example, the ability to conceptualize and formulate scientific hypotheses and test them during experimental data collection. During the research internship, URM scholars work with experienced STEM URM researchers who serve as mentors in the program. This mentorship provides guidance, support, and advice from mentors who experience similar URM challenges and provide insight into overcoming challenges. The research mentors provided one-on-one training in various laboratory techniques, which helped URMs develop skills in critical thinking, problem-solving, and data analysis. They implemented rigorous scientific research projects and presented their findings at different national conferences. Presenting at research conferences likely increased the confidence in our URM student scholars and validated their abilities in performing scientific research to enrich their science identity, and this includes knowledge of the STEMM discipline, ability to perform relevant scientific research, as well as a sense of belonging and recognition within a scientific network (Carlone & Johnson, 2007). This is because URMs experienced the collaborative and empowering culture of science through collaborating work, and exhibited strong science identities as a result of conducting scientific research during the research internship activities of the REP program. Moreover, presenting their work at regional and national conferences increased their confidence to respond to questions related to their research and also ask questions and constructively challenge ideas or approaches related to experimental designs that promote readiness to become independent in conducting research. The outcome is greater participation in research activities and stronger research performance during their internship program. This is reflected in the numerous abstracts accepted and presented at national conferences and publications. These experiences occur within the context of a cohort of scholars who are intentionally provided with opportunities to build community both within the cohort and within the broader research environment at our institution, local, and national conferences, and institutions participating in the program. Through this program, scholars gained increased self-efficacy in a spectrum of competencies, thereby fostering greater science identity and a heightened inclination to pursue and persevere in scientific research careers.

Our finding is supported by other studies that involvement in internships may allow URM students to feel like they are contributing meaningfully to the field (Barongan et al., 2023; Kricorian et al., 2020), help students learn the norms of science (Marriott et al., 2021; Massey et al., 2022), and decrease feelings of marginalization by integrating the student into the STEM community (Bowman et al., 2023; Moreira et al., 2019; Nelson et al., 2023). We observed that all the research mentors were either very satisfied or satisfied with their student interns' ability to balance research with academic work during the internship. We collected feedback from URM students about their experiences and actively incorporated their input into the design and implementation of the internship program. This allowed each mentor to tailor the research internship activities with flexibility such that students could work at their own pace and available time without affecting class schedules. Therefore, the design of the research internship program was well-integrated with the academic goals of each URM student and with mentorship, that aligns with students' interests, and they gained academically. The addition of stipends to address systemic financial barriers to success further motivated URM students to fully participate in the research internship program. A similar finding has been shown by other studies (Matthews et al., 2022; Stephenson-Hunter et al., 2021). Findings from these studies indicate that financial support for URMs has a positive influence on student persistence. Indeed, one of the most compelling factors affecting the supply of minority STEM graduates has involved financial incentives for research internships (Edwards et al., 2023a, b; Pender et al., 2010). This is because offering stipends can play a major role in addressing systemic financial barriers that may hinder URMs from fully participating in research internship programs (Edwards et al., 2023a, b; Ives et al., 2023). Our current results indicate that such stipends can make a difference in URM students who come from backgrounds where financial resources are limited by ensuring that these students can afford necessities such as food and transportation during the internship period. This removes a significant barrier to participation in research programs and promotes success among URM students in research internship programs (Edwards et al., 2024; Wilson et al., 2018).

Promoting STEMM education and fostering robust science identities as well as knowledge of STEMM disciplines, particularly among diverse groups, is essential for cultivating a future-ready workforce and addressing global challenges. Our strategy focuses on enhancing STEMM education through hands-on learning experiences provided by our research internship program. We ensure that URM students have access to laboratories, equipment, and valuable research opportunities, while also pairing them with trained STEMM mentors and diverse role models to inspire them in their scientific endeavors. Therefore, our research internship program immerses URM students in real-world STEMM applications, allowing them to engage in meaningful scientific research. We enhance their research skills by offering training in essential research methods and providing hands-on experiences in data analysis and interpretation. In addition, we provide funding to ensure that URM research interns present their research findings at national and regional conferences. Additionally, we guide them in drafting research papers and conference abstracts. Research mentors make it a priority to recognize the contributions and progress of these students, highlighting their strengths, building their confidence, promoting self-efficacy, and encouraging them to take ownership of their work. We celebrate their achievements during lab meetings, especially when their abstracts and papers are accepted or presented at conferences. Moreover, we provide students with resources for their research and opportunities to present their findings at national and regional conferences. Participation in these conferences helps underrepresented students build networks within scientific communities, boosts their confidence, expands their opportunities, and fosters a sense of belonging in the STEMM field.

Our finding was that all the students were satisfied with how the internship program improved their professional development in scientific research, oral presentation skills, and written communication skills through the preparation of their posters and manuscripts. This program also enhanced their ability to work as a productive team member. This is also reflected in the positive correlation between student satisfaction and research mentor evaluation of their satisfaction with students' performance during the research internship program. Overall, our research internship program for URM students was successful in providing URM students with a wide-ranging professional development experience that not only improved their scientific research skills but also enhanced their ability to communicate their findings effectively, both orally and in writing, and to collaborate with others in a team setting. These skills are invaluable for success in both academic and professional settings and can help URM students overcome barriers and achieve their career goals in STEM fields.

## Limitation

Findings from this study are limited to the outcomes of one STEM research internship program from one institution, therefore results may not be generalized to students from different geographic areas or different types of programs. Furthermore, although we analyzed quantitative data for both research mentorship and interns' perceptions of the internship program, we did not collect qualitative data to help provide a broader evaluation of both students' and research mentors' satisfaction. Findings from this study present important outcomes associated with the importance of integrating research internship programs in REP beyond summer activities and contribute to the extant literature by addressing the key elements that could significantly increase URM students’ aspirations for STEMM graduate programs.

## Conclusion

Findings from this study underscore the importance of research internship programs for URM students. Given the strong empirical evidence supporting the significance of research internships in strengthening the readiness of URM students for research-intensive STEMM graduate programs, success, and persistence in STEMM careers, earlier interventions to recruit and prepare URM students to enter and succeed in the STEM workforce is imperative. Findings from this study underscore the importance of early intervention with research internship programs as an essential foundation for establishing minority students’ science identity and integration into their scientific community. Our findings support existing literature that highlights the importance of research internships in laying the groundwork for URM students to develop the academic skills, resiliency, and motivation to succeed in an increasingly challenging academic environment. Given these findings, further work is needed to determine how research internship programs can strengthen collaborative learning relative to early exposure to research and how this may precisely influence student success factors. Such information could provide valuable recommendations to program administrators on how to improve and modify program components in ways that are particularly helpful to URM populations and address their specific challenges in REP.

## Data Availability

Materials are available on request from the corresponding author.
